# Joint EURADOS-EANM initiative for an advanced computational framework for the assessment of external dose rates from nuclear medicine patients

**DOI:** 10.1186/s40658-024-00638-y

**Published:** 2024-04-22

**Authors:** Lara Struelens, Christelle Huet, David Broggio, Jérémie Dabin, Laurent Desorgher, Augusto Giussani, Wei Bo Li, Dietmar Nosske, Yi-Kang Lee, Lidia Cunha, Maria J. R. Carapinha, Mario Medvedec, Peter Covens

**Affiliations:** 1 Belgian Nuclear Research Center (SCK CEN), Nuclear Medical Applications, Boeretang 200, 2400 Mol, Belgium; 2 Institut de Radioprotection et de Sûreté Nucléaire (IRSN), PSE-SANTE/SDOS, 31 Avenue de La Division Leclerc, 92260 Fontenay-Aux-Roses, France; 3 Institute of Radiation Physics, Lausanne University Hospital and University of Lausanne, Lausanne, Switzerland; 4 Federal Office for Radiation Protection (BfS), Ingolstädter Landstr. 1, 85764 Oberschleißheim, Germany; 5 Université Paris-Saclay, CEA, Service d’études des réacteurs et de mathématiques appliquées, 91191 Gif-Sur-Yvette, France; 6 Nuclear Medicine and Molecular Imaging, IsoPor-Azores, Canada do Breado, 9700 Angra Do Heroismo, Azores Portugal; 7 ESTeSL-Escola Superior de Tecnologia da Saúde, Instituto Politécnico de Lisboa, Lisboa, Portugal; 8 Department of Nuclear Medicine and Radiation Protection, University Hospital Centre Zagreb, Zagreb, Croatia; 9 Molecular Imaging and Therapy, Vrije Universiteit Brussel (VUB), Brussels, Belgium

**Keywords:** Nuclear medicine patients, Caregivers, Release criteria, Monte Carlo, Computational models

## Abstract

**Background:**

In order to ensure adequate radiation protection of critical groups such as staff, caregivers and the general public coming into proximity of nuclear medicine (NM) patients, it is necessary to consider the impact of the radiation emitted by the patients during their stay at the hospital or after leaving the hospital. Current risk assessments are based on ambient dose rate measurements in a single position at a specified distance from the patient and carried out at several time points after administration of the radiopharmaceutical to estimate the whole-body retention. The limitations of such an approach are addressed in this study by developing and validating a more advanced computational dosimetry approach using Monte Carlo (MC) simulations in combination with flexible and realistic computational phantoms and time activity distribution curves from reference biokinetic models.

**Results:**

Measurements of the ambient dose rate equivalent Ḣ^*^(10) at 1 m from the NM patient have been successfully compared against MC simulations with 5 different codes using the ICRP adult reference computational voxel phantoms, for typical clinical procedures with ^99m^Tc-HDP/MDP, ^18^FDG and Na^131^I. All measurement data fall in the 95% confidence intervals, determined for the average simulated results. Moreover, the different MC codes (MCNP-X, PHITS, GATE, GEANT4, TRIPOLI-4*®*) have been compared for a more realistic scenario where the effective dose rate *Ė* of an exposed individual was determined in positions facing and aside the patient model at 30 cm, 50 cm and 100 cm. The variation between codes was lower than 8% for all the radiopharmaceuticals at 1 m, and varied from 5 to 16% for the face-to face and side-by-side configuration at 30 cm and 50 cm. A sensitivity study on the influence of patient model morphology demonstrated that the relative standard deviation of Ḣ^*^(10) at 1 m for the range of included patient models remained under 16% for time points up to 120 min post administration.

**Conclusions:**

The validated computational approach will be further used for the evaluation of effective dose rates per unit administered activity for a variety of close-contact configurations and a range of radiopharmaceuticals as part of risk assessment studies. Together with the choice of appropriate dose constraints this would facilitate the setting of release criteria and patient restrictions.

## Background

The growing number of nuclear medicine (NM) procedures and the diversity of new radiopharmaceuticals, stimulate the need for a more comprehensive re-evaluation of the radiological risk of staff, caregivers and the general public coming into proximity of NM patients (which will be described in the entire paper as ‘critical groups’). In order to ensure adequate radiation protection of these critical groups, it is necessary to consider the impact of the radiation emitted by the patients during their stay at the hospital or after leaving the hospital. Radiation exposure of medical staff is well documented because of routine individual monitoring programs [2] and numerous optimization studies [19]. The potential radiation exposure of caregivers and the general public after release of the NM patient from the hospital is generally less documented and relies on specific risk assessment studies that are the basis for setting release criteria and patient restrictions. In case of therapeutic procedures, several studies report data on the potential exposure of these critical groups for ^131^I therapy to treat thyroid cancer or hyperthyroidism, and today’s patient release criteria and restrictions are mainly related to these types of procedures. In case of diagnostic procedures, risk assessment studies indicate no need of any restrictions once the patient is released from the hospital although some restrictions are still recommended in radiopharmaceutical leaflets. Today, recommendations regarding release of the NM patient after both therapeutic and diagnostic NM procedures vary widely around the world. Some important factors that lead to this variation are differences in recommended dose constraints for specific critical groups and the different applied methods in risk assessment studies [12].

Risk assessment studies for both diagnostic and therapeutic procedures are based on ambient dose rate measurements in a single position at a specified distance from the patient and carried out at several time points after administration of the radiopharmaceutical to estimate the whole-body activity retention. As such, these external dose rates are estimated by making a series of simplifications for modelling, both the radiation emitted by the patient and the dose to an individual of a critical group. To assess the exposure level, these ambient dose rate measurements are then combined with specific exposure scenarios, describing how long individuals are in close contact with the patient over time. On the one hand, these approximations simplify the dose assessments task, but on the other hand they also affect their accuracy. Since the patient is a physically large and inhomogeneous radiation source with varying activity distribution over time, reducing that source to a dose rate in a single point is prone to lead to large errors in dose estimations, especially in close contact scenarios. In order to address the current limitations, a more comprehensive advanced dose calculation approach was developed by using Monte Carlo simulations in combination with the most recent advancements in computational dosimetry, i.e. realistic flexible anthropomorphic phantoms [11, 17], and time activity distribution curves from reference biokinetic models [6, 10]. In this paper, we present a computational framework and its validation for external dose rates from NM patients administered with ^99m^Tc-HDP/MDP, ^18^FDG and Na^131^I. This study is a collaboration between the European Radiation Dosimetry Group (EURADOS) and the Radiation Protection Committee of the European Association of Nuclear Medicine (EANM). This collaboration allowed us to benchmark the computational approach against experimental data and between different Monte Carlo codes.

## Material and methods

**Fig. 1:**
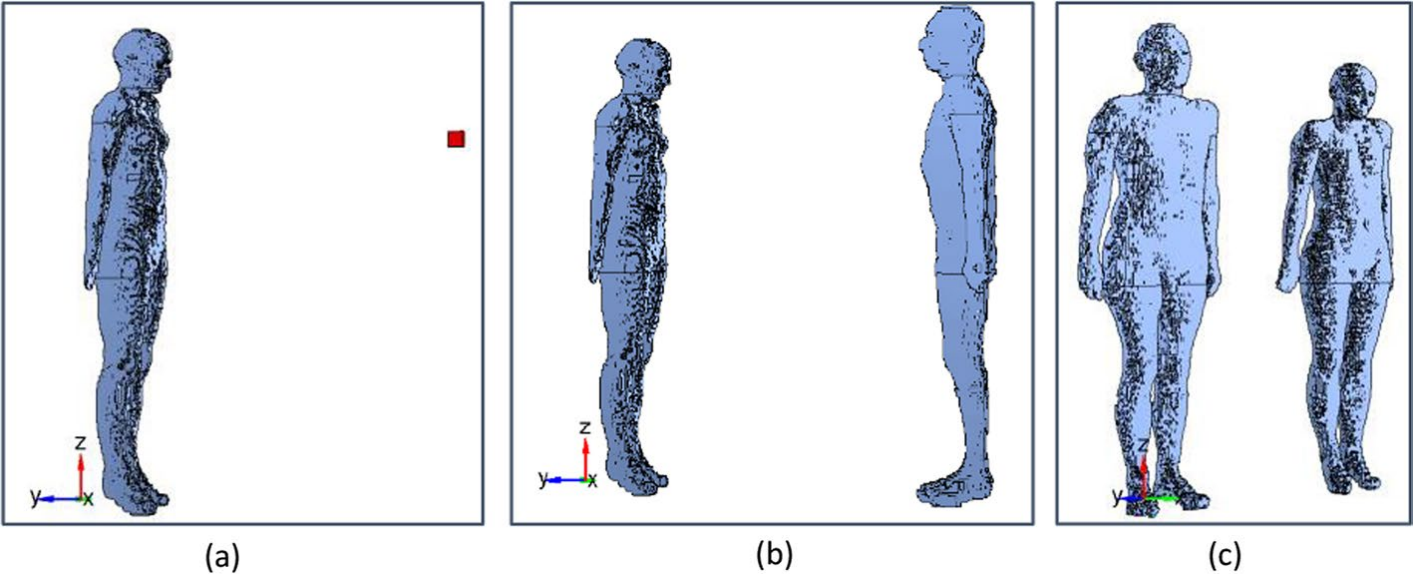
MC geometry of Ḣ^*^(10) in a point (**a**), *Ė* for a model of an exposed individual, facing (**b**) or aside (**c**) the NM patient. The NM patient model is represented by the ICRP reference female computational voxel model [9]. The exposed individual is represented alternatively by the ICRP reference female and male (only shown in the figure) computational voxel models to calculate sex-averaged effective doses

Three different configurations have been considered in this benchmarking study (Fig. 1):

- The ambient dose rate equivalent Ḣ^*^(10) was calculated 1 m away from the NM patient, represented by the reference female computational phantom of the International Commission on Radiological Protection (ICRP) [9]. For ^99m^Tc (who has the lowest gamma emission energy of 140 keV from the 3 isotopes considered), Ḣ^*^(10) was also calculated at 30 cm and 50 cm from the patient (Fig. 1a).
- The effective dose rate *Ė* of an individual of a critical group was determined in a position facing the patient model at 1 m. For ^99m^Tc, *Ė* was also calculated at 30 cm and 50 cm from the patient (Fig. 1b).
- The effective dose rate *Ė* of an individual of a critical group was determined in a position aside the patient model at 1 m. For ^99m^Tc, *Ė* was also calculated at 30 cm and 50 cm in this configuration (Fig. 1c).

The benchmarking study was performed for typical clinical procedures: ^99m^Tc-HDP/MDP (Hydroxymethylene diphosphonate /Methylene diphosphonate), ^18^FDG (2-Deoxy-2-[18F]fluoroglucose) and Na^131^I (Sodium iodide).

### Biokinetic models and time activity curves for ^99m^Tc-HDP/MDP, ^18^F-FDG and Na^131^I

For ^99m^Tc-HDP/MDP, the biokinetic data early published in ICRP Publication 53 [6] and later adopted by ICRP Publication 128 [10] was used in this study. The time activity curves in the source regions (Table 1) were calculated analytically using biokinetic data. Urinary excretion in the bladder is calculated separately according to Snyder and Ford [22]. Also for Na^131^I, the current ICRP biokinetic model in Publication 128 [10] was used in the present work for the source regions presented in Table 1. The compartmental model for ^18^FDG which was recently developed by an ICRP task group [15] was applied. The time activity curves in the source regions indicated in Table 1 were calculated using the software SAAM II (Nanomath (LLC), Spokane, WA, USA). To model the urinary excretion rate of patients in a realistic way, for these three given radiopharmaceuticals, the information of patient bladder voiding time obtained from the ambient dose rate measurement data at 1 m from NM patients was used in the modelling. These measurement data are described in one of the following subsections of this paper.

**Table 1:** Radiopharmaceuticals and source regions

Radiopharmaceutical	Source regions
^99m^Tc-HDP/MDP	Urinary bladder content, kidneys, bone and the remainder
^18^FDG	Urinary bladder content, kidneys, liver, blood, brain, hearth, lungs, pancreas, spleen and the remainder
Na^131^I	Urinary bladder content, kidneys, liver, blood, salivary glands, stomach wall, thyroid, small intestine content, right, left and recto-sigmoid colon content and the remainder

For the 3 given radiopharmaceuticals, calculations of the time activity curves in corresponding source regions were performed independently by different groups of scientists and benchmarked against each other. For ^99m^Tc-HDP/MDP and Na^131^I it was evaluated if the integrals of time activity curves were in agreement with the ICRP values.

### Monte Carlo simulations

In the Monte Carlo simulations, the organs of the patient model are defined as radioactive source regions, while the tissues of the exposed individual model(s) are defined as target tissues, i.e. tissues used to score energy depositions and thus their absorbed doses. For the source modelling, the biokinetics of the ^99m^Tc-, ^18^F- and ^131^I-based radiopharmaceuticals in the different source regions are considered.

The effect of each source organ on the exposed individual is additive, therefore the simulations are performed for each source region individually. The output of the MC codes is given in Sv per emitted source particle. By considering the total emission probabilities of a given radionuclide (i.e. the number of particles emitted per second), simulated doses are converted to Sv/s/Bq. Finally, all results in this study are expressed in µSv/h/MBq.

- Depending on the participating MC team, Ḣ^*^(10) is determined by
  - calculating the fluence in air in a point (represented as a 1 mm^3^ cube) positioned at chest height of the patient model at 1 m from the patient’s skin. Fluence is converted into Ḣ^*^(10) by using the energy-dependent conversion coefficients from ICRP-74 [7].
    or
  - calculating the deposited energy at 10 mm depth of an ICRU (International Commission on Radiation Units and Measurements) sphere located at 1 m from the patient’s skin at chest height, according to Ḣ^*^(10) definition.

When summing the results over all source regions, the resulting cumulated Ḣ^*^(10) for each time point (t) post-administration, is given by Eq. 1:

1 $$\dot{H}^{*} \left( {10} \right)\left( t \right) = \sum_{{{\text{source}}\;{\text{ region}}}} {a_{{{\text{source }}\;{\text{region}}}} \left( t \right)\dot{H}^{*} \left( {10} \right)_{{{\text{source}}\;{\text{ region}}}} }$$

With

- Ḣ^*^(10)(*t*): ambient dose rate equivalent per unit of administered activity at time t post administration (Sv/s/Bq).
- a_source region_(t): fractional activity of the administered activity in the source region at time t post administration.
- Ḣ^*^(10)_source region_: ambient dose rate equivalent per unit of administered activity in the source region (Sv/s/Bq)

*Ė(t)* of an individual is obtained as the average of the organ dose rates to both the male and female ICRP reference voxel phantoms, weighted with the ICRP-103 tissue weighting factors [8]. The cumulated effective dose rate for each time point (t) post-administration per administered activity is given by Eq. 2:

2 $$\dot{E}\left( t \right) = \sum\limits_{{{\text{source }}\;{\text{region}}}} {a_{{{\text{source }}\;{\text{region}}}} \left( t \right)\dot{E}_{{{\text{source}}\;{\text{ region}}}} }$$

With

- *Ė(t)*: Effective dose rate per unit of administered activity at time t post administration (Sv/s/Bq)
- a_source region_(t): fractional activity of the administered activity in the source region at time t post administration
- Ė_source region_: effective dose rate per unit of administered activity in the source region (Sv/s/Bq)

The Monte Carlo simulations for both set-ups are performed with five different radiation transport codes: PHITS (version 3.2) [24], MCNP-X (version 2.6c) [26], TRIPOLI-4*® *[5], Geant-4 (version 11.0) [1] and GATE (version 9.2) [14] (only for ^131^I). The results from all codes are compared for both configurations, separately per source organ and for the cumulated Ḣ^*^(10) or *Ė* over time.

### Ambient dose rate measurements

In seven different European NM departments, Ḣ^*^(10) measurements were performed at 1 m from the patients at different time points post-administration, with two measurements for each time point. Measurement data was obtained in six hospitals for ^99m^Tc-HDP/MDP, in three hospitals for ^18^FDG, and in one hospital for Na^131^I. First, an intercomparison exercise of the measurement equipment was performed for ^99m^Tc-HDP/MDP and ^18^FDG. In each participating hospital Ḣ^*^(10) were measured three times at 1 m from a 10 ml vial containing an activity calibrated with the activity meter of the local radiopharmacy laboratory. The calibrated measurement equipment of the participating hospitals is listed in Table 2, one of the three hospitals that performed measurements for ^18^FDG did not participate to this intercomparison exercise. The measurements are compared against a reference value, determined through Monte Carlo simulations. The fluence in a 1 mm^3^ air cube at 1 m from the center of the simulated vial is calculated and converted to Ḣ^*^(10) with the ICRP74 conversion coefficients [7]. The vial is modelled as a 50 mm high cylinder with 20 mm diameter and 1 mm wall thickness. The walls are made of borosilicate glass and the vial is half-filled with a water solution, containing the radioactive source.

**Table 2:** Measurement equipment for Ḣ^*^(10) dose rate measurements

Hospital	Brand, model	Detector type	Patient measurements
A	Victoreen/Fluke/Innovision 451B	Ionization chamber	^99m^Tc-HDP/MDP, ^18^FDG
B	Berthold LB123Umo + LB1236-H10	Proportional counter	^99m^Tc-HDP/MDP
C	ATOMTEX AT1125	Geiger-Mueller	^99m^Tc-HDP/MDP, ^18^FDG, Na^131^I
D	Victoreen/Fluke/Innovision 451P	Ionization chamber	^99m^Tc-HDP/MDP
E	AUTOMESS S 6150 AD4	Geiger-Mueller	^99m^Tc-HDP/MDP
F	Canberra Radiagem 2000	Geiger-Mueller	^99m^Tc-HDP/MDP

For the ambient dose rate measurements at 1 m from the patients in the participating hospitals, patient (sex and BMI) and procedure data (radiopharmaceutical, administered activity, syringe/vial residual activity after administration, number and timing of bladder voiding, if applicable) are collected. The measurement data of each patient is corrected for the difference against the nominal value obtained from the equipment intercomparison exercise and compared against the Monte Carlo simulations performed for the set-up illustrated in Fig. 1a. For this comparison, the average of Ḣ^*^(10)_source organ_ from the simulation results of all participating Monte Carlo codes was taken.

### Sensitivity study on the influence of patient model morphology

To evaluate the influence of body morphology on the external dose rates from NM patients, a sensitivity study is performed calculating the ambient dose rate equivalent Ḣ^*^(10) at 1 m from four different computational voxel phantoms, selected from a phantom library developed by Broggio et al. [3]. Four male models are selected with a model height of 164 ± 6 cm, which is close to the height of the ICRP reference female voxel phantom used in the previous simulations, and a body mass ranging from 50 to 85 kg (Table 3).

**Table 3:** Body morphology of computational models used for the sensitivity study

Phantom code^*^	Height (cm)	Weight (kg)
M0A	162.7	50.8
M0B	164	60.2
M0C	164	71.9
M0D	165.5	85.3

^*^Phantom code from [3]

The Monte Carlo simulations are performed with MCNPX 2.6c for the radionuclide ^99m^Tc.

### Uncertainty assessment

The following contributions to the total uncertainty on the experimental data of ambient dose rate equivalent per administered activity at 1 m from the patient have been considered:

- The uncertainty of the ambient dose rate measurement device used by the specific center. This uncertainty is mainly defined by the energy and angular response from the device itself and is a priori unknown for the devices used in this study. Therefore, the average relative deviation of the different measurement devices used in the intercomparison exercise against the reference value for ^99m^Tc is used to represent this device-related uncertainty contribution.
- The positioning uncertainty of the measurement device, caused by the different sizes of the sensitive volume of the chambers used. Assuming a possible error of 10 cm in both directions along the line patient-detector at their distance of 1 m, this results in an uncertainty of ± 20%, applying the inverse square law.
- The uncertainty on the activity measurement of the syringe used for patient administration. From previous national and international intercomparison studies of radionuclide calibrators [20, 21]

For the uncertainty on the simulated Ḣ^*^(10)(*t*) data, the following contributions are considered:

- The absolute uncertainty on the mean value obtained from all the different MC codes for the Ḣ^*^(10)_source organ_ factor in (Eq. 1) for each individual source organ.
- The uncertainty on the activity values per time point for each source organ as a result of the biokinetic model parameters. Although this uncertainty is expected to be the largest of all uncertainty contributions in this study, it is very difficult to quantify it. In general, a variability by a factor of 2 or more is considered reasonable for kinetics of any given radiopharmaceutical as stated by [23] and variations of the same order of magnitude can be observed in whole-body retention curves from [16, 18] for ^177^Lu-based therapies. With time-activity curves considered as being log-normal, a factor 2 variability means that the activity values lie within [A(t)/2 and 2xA(t)).
- The statistical uncertainty on the simulated results for each code was very small (< 1%) and therefore neglected in the total uncertainty assessment.

Following the GUM (Guide to the expression of Uncertainty in Measurement) approach [13] for combined uncertainties for quantities obtained following Eq. 1, the total uncertainty U(t) on the simulated Ḣ^*^(10) data is obtained as follows:

3 $$U\left( t \right) = \left( {\left[ {a_{1} \left( t \right)*u\left( {C_{1} } \right)} \right]^{2} + \left[ {a_{2} \left( t \right)*u\left( {C_{2} } \right)} \right]^{2} + \ldots + \left[ {C_{1} *u\left( {a_{1} \left( t \right)} \right]^{2} + } \right[C_{2} *u\left( {a_{2} \left( t \right)} \right]^{2} + \ldots } \right)^{{{\raise0.7ex\hbox{$1$} \!\mathord{\left/ {\vphantom {1 2}}\right.\kern-0pt} \!\lower0.7ex\hbox{$2$}}}}$$

with u(C_i_) the uncertainty on Ḣ^*^(10)_source organ_ for source organ *i* and u(a_i_(t)) the uncertainty on the fractional activity in source organ *i* at time point *t*. In order to be able to apply the uncertainty propagation formula in Eq. 3, the log-normal uncertainty factor of 2 needs to be transformed into an uncertainty factor describing a normal distribution in the following way: $u\left( {a_{i} \left( t \right)} \right) = a\left( t \right)\left[ {\exp \left[ {\left( {\ln \left( 2 \right)/k} \right)^{2} } \right] - 1} \right]^{{1/2}}$ using the coverage factor *k* = 2 for 95% confidence intervals [4].

## Results

### Comparison of Monte Carlo codes

**Fig. 2:**
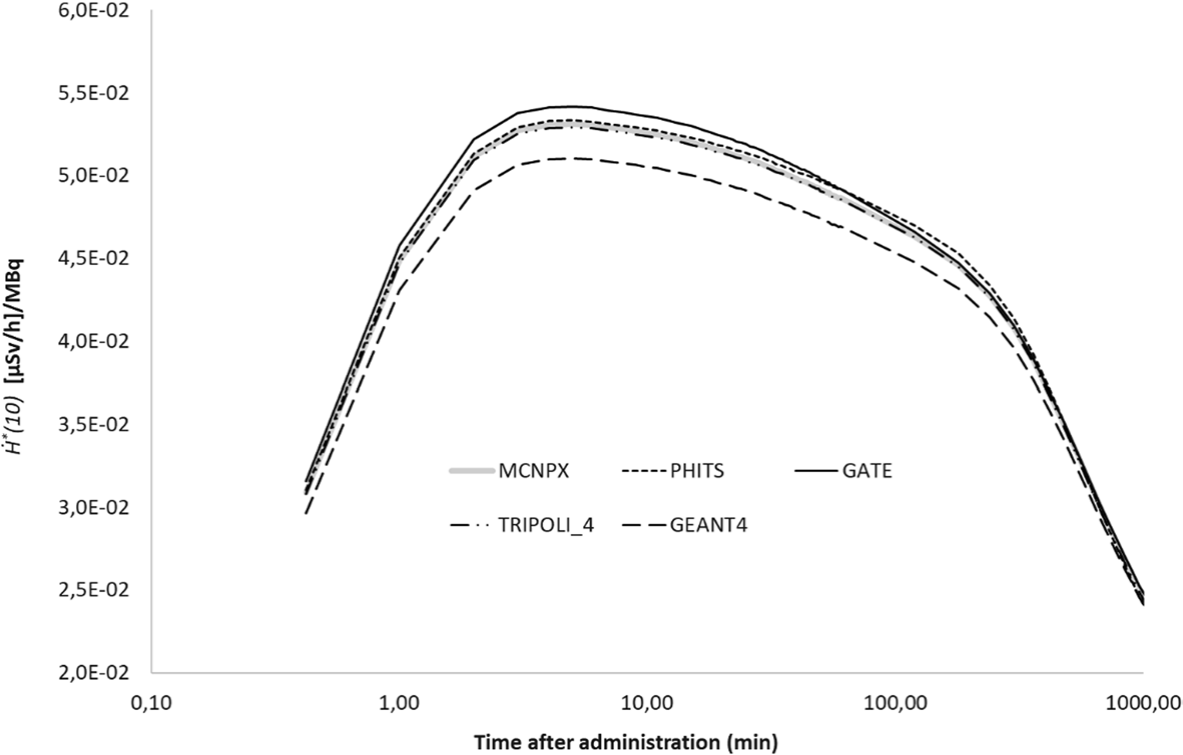
Simulation data of five MC codes for Ḣ^*^(10)(t) at 1 m from patient model for Na^131^I. The NM patient model is represented by the ICRP reference female computational voxel model

In Fig. 2, the Ḣ^*^(10)(t) at 1 m from the patient is shown for the five MC codes for Na^131^I. The variation between the different codes is less than 5% for all the different source regions. For ^99m^Tc-HDP/MDP the variation between the different codes ranges from 5 to 10% for the different source regions, with the highest variation observed with the bone as source region. For ^18^FDG the variation between the codes is less than 4% for all source regions.

**Fig. 3:**
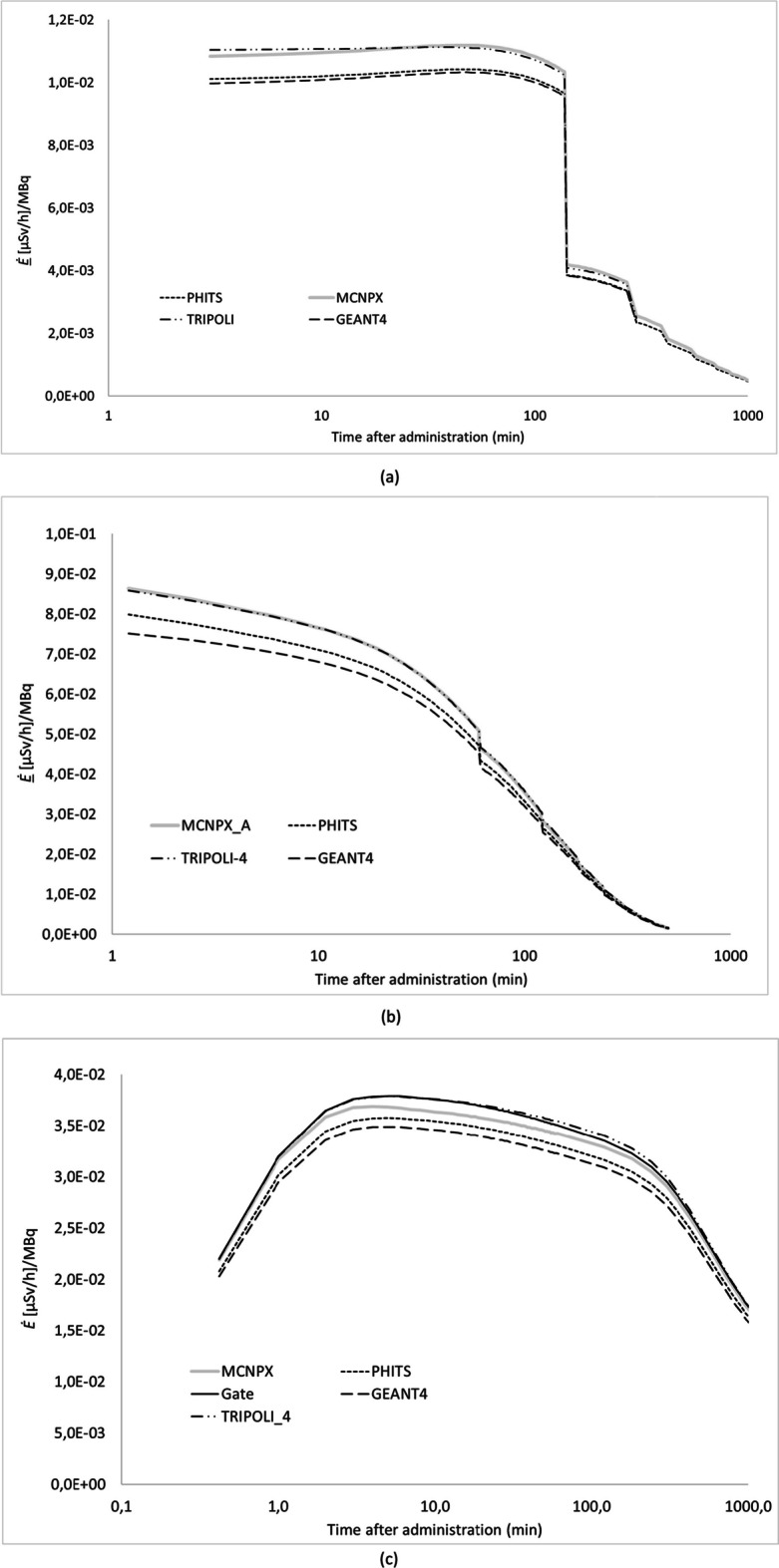
Simulation data of four/five MC codes for *Ė(t)* at 1 m from patient model for ^99m^Tc-HDP/MDP (**a**), ^18^FDG (**b**) and Na^131^I (**c**). The NM patient model is represented by the ICRP reference female computational voxel model. The exposed individual is represented alternatively by the ICRP reference female and male computational voxel models to calculate sex-averaged effective doses

**Fig. 4:**
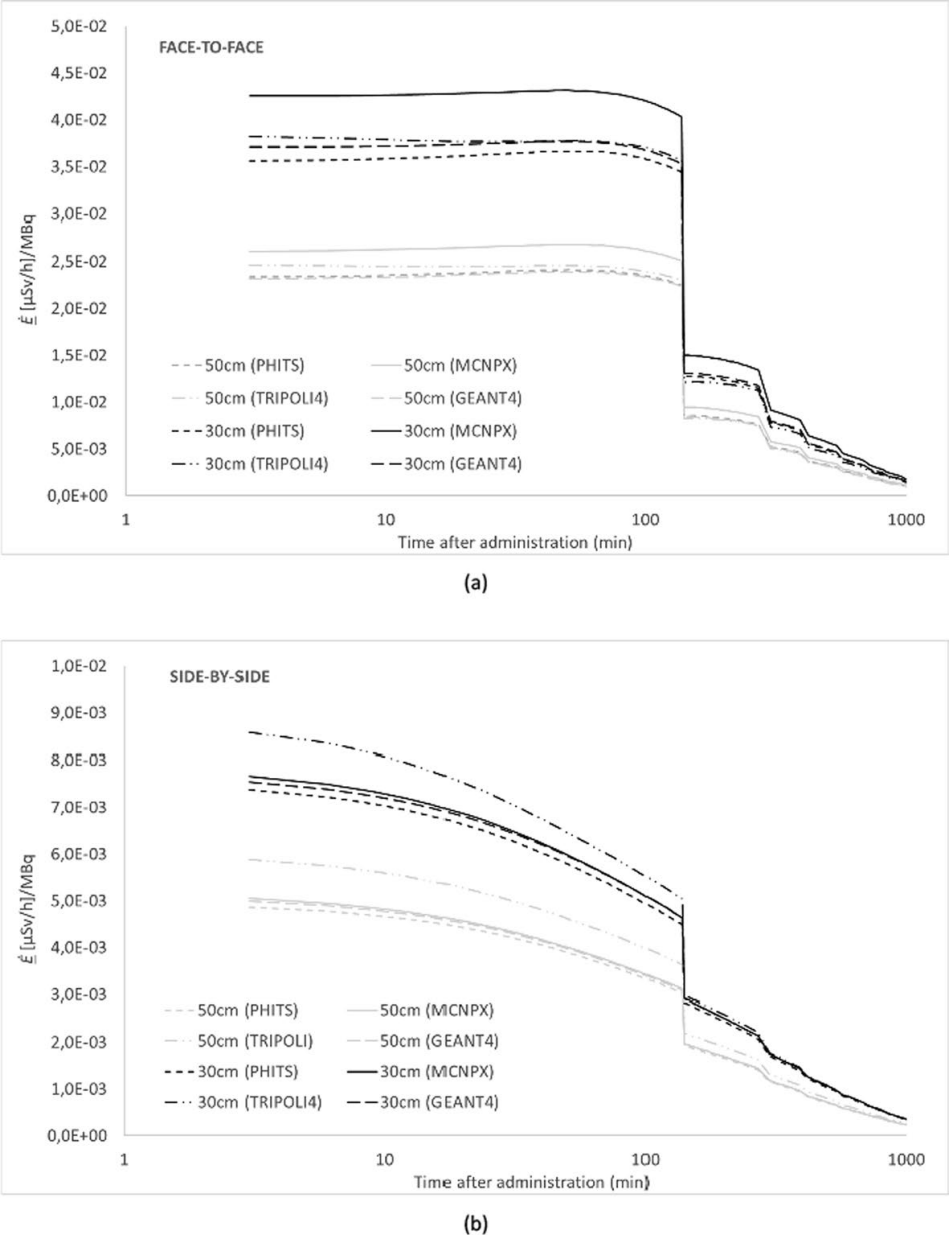
Simulation data of four MC codes for *Ė(t)* for ^99m^Tc-MDP/HDP with the computational phantoms facing each other at 30 cm and 50 cm (**a**) and side by side at 30 cm and 50 cm (**b**) The NM patient model is represented by the ICRP reference female computational voxel model. The exposed individual is represented alternatively by the ICRP reference female and male computational voxel models to calculate sex-averaged effective doses

In Fig. 3, *Ė(t)* is compared between four or five (depending on the radiopharmaceutical) MC codes, with both the patient and the exposed individual represented as ICRP voxel phantoms facing each other at 1 m distance for ^99m^Tc, ^18^F and ^131^I, respectively. The variation between the codes was lower than 8% for all the different source regions and three given radionuclides. Figure 4 shows the effective dose rates *Ė(t)* per code for the face-to face configuration (Fig. 4a) and the side-by-side configuration (Fig. 4b) at 30 cm and 50 cm, respectively. On average, the difference between the Monte Carlo codes varies from 5 to 16% for the considered source regions.

### Comparison against experimental data

**Fig. 5:**
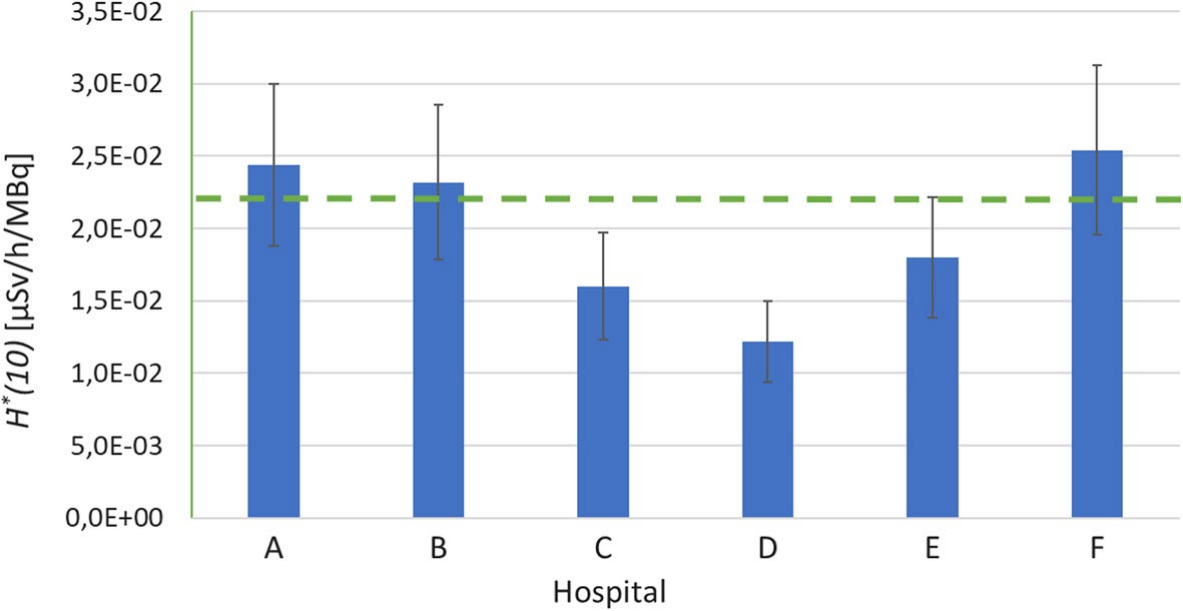
Comparison of measurement equipment of different hospitals for the measurement of ambient dose rate Ḣ*(10) at 1 m from a ^99m^Tc vial. The green line is the simulated reference value of 2.21E-02 µSv/h/MBq

Figure 5 shows the Ḣ^*^(10) results of the device intercomparison exercise from the participating hospitals against the simulated reference value of 2.21E-02 µSv/h/MBq for ^99m^Tc. An average relative deviation of 10% against the reference value was obtained, with a maximum deviation of -45% for center D.

In Fig. 3a, the fast drop in Ḣ^*^(10)(t) at 210 min post administration (p.a.) for ^99m^Tc-HDP/MDP corresponds to the voiding of the bladder according to the standard assumptions used in ICRP-128 [10]. For the measurement data with ^99m^Tc-HDP/MDP in the hospitals, patients are requested to empty their bladder prior to image acquisition, which is typically scheduled on average 120 min p.a. The recorded patient data shows that time of bladder voiding ranged from 27 min p.a. to a maximum of 234 min p.a. (interquartile range: Q1 = 91 min; Q3 = 157 min). As the standard bladder voiding time of 210 min p.a. from ICRP-128 was far off from the typical patient data, for comparison to the measurements, the biokinetic model for the urinary bladder has been adjusted to typical bladder voiding times observed in the involved patient population as follows:

- Bladder voiding at 30 min p.a. and every 1 h afterwards
- Bladder voiding at 45 min p.a. and every 1 h afterwards
- Bladder voiding with 120 min interval p.a.
- Bladder voiding time with 210 min interval p.a. (ICRP-128 biokinetic model)

**Fig. 6:**
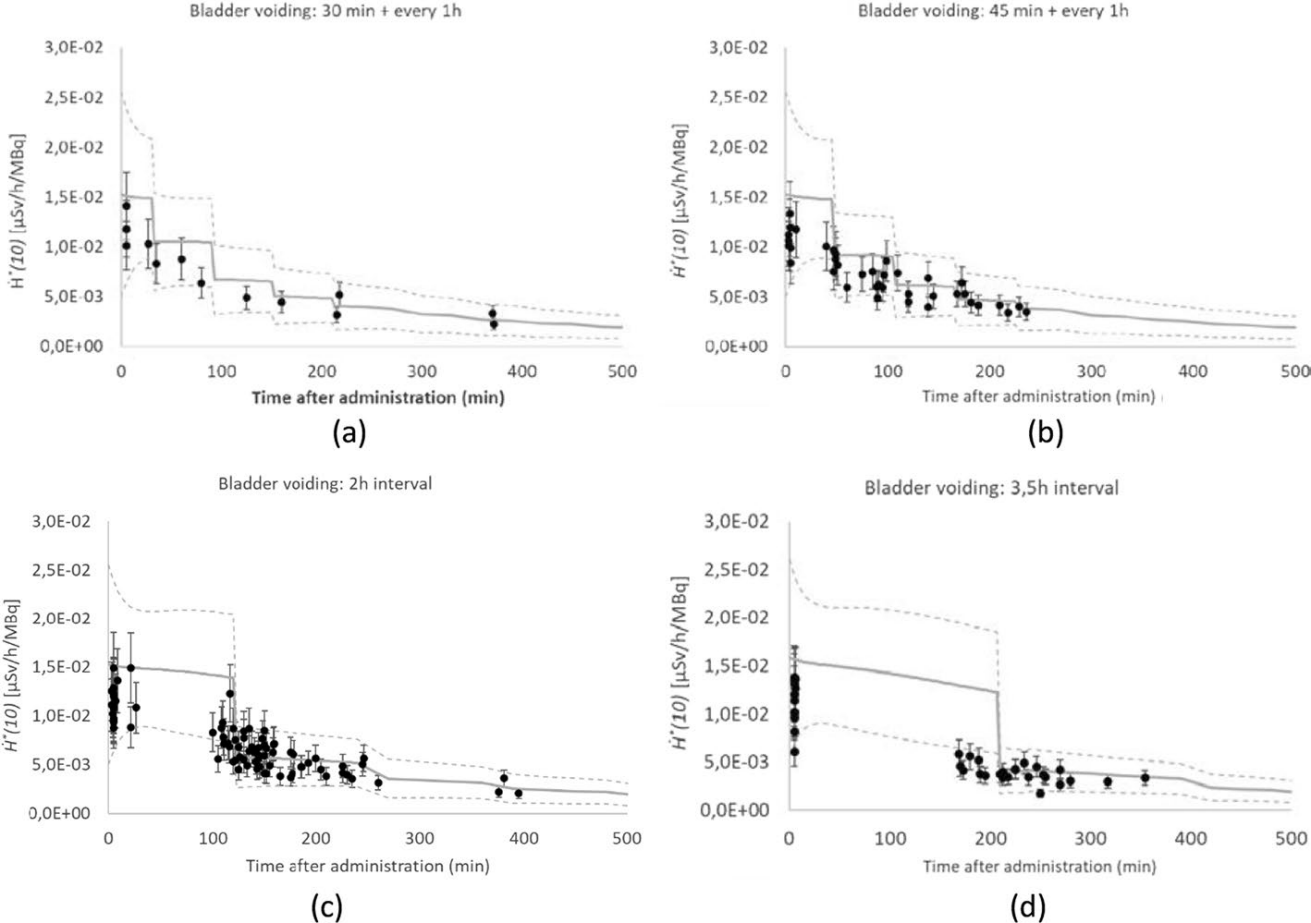
Comparison of simulated (solid line) and experimental (dots) Ḣ^*^(10)(t) for patients injected with ^99m^Tc-HDP/MDP. Data is categorized (**a**-**d**) according to the bladder voiding time p.a. of the patient population and the biokinetic model of the bladder adapted accordingly

**Fig. 7:**
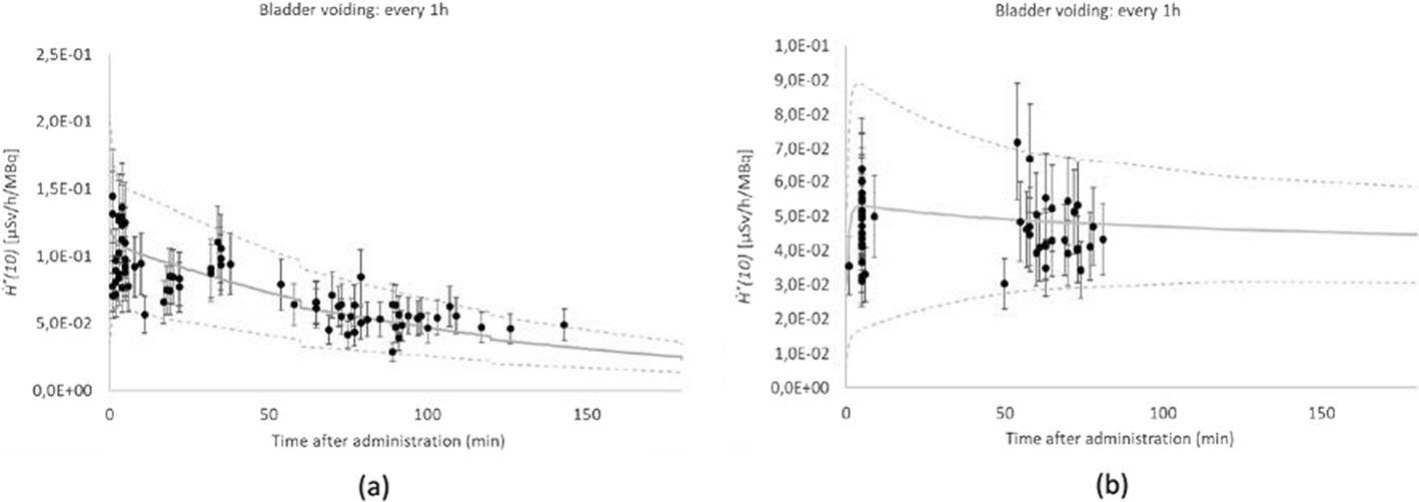
Comparison of simulated (solid line) and experimental (dots) Ḣ^*^(10)(t) for patients, administered with ^18^FDG (**a**) and Na.^131^I (**b**)

Next, the experimental Ḣ^*^(10) values per unit administered activity of ^99m^Tc-HDP/MDP measured at 1 m from the patients was categorized in one of the abovementioned categories according to the patient-specific first bladder voiding time. The comparison was made against the simulated data (average from all MC codes), using for each category the biokinetic model with similar bladder voiding regime (Fig. 6). After taking into account the patients’ bladder voiding time, a good agreement was found between the simulated and experimental data. The error bars on the experimental data represent a total combined (*k* = 2) uncertainty of 24% for the ambient dose rate equivalent per unit activity measurement at 1 m of patients administered with ^99m^Tc-HDP/MDP, ^18^FDG or Na^131^I. The combined uncertainty includes the device-specific uncertainty (10%), the positioning uncertainty (10%) and the uncertainty on the activity measurement (5–10%, depending on radiopharmaceutical), as described in the Material and Methods section. The dashed lines in Fig. 6, represent the uncertainties on the simulated data, given as 95% confidence intervals, determined by Eq. 3. In Fig. 7, the experimental Ḣ^*^(10) data are compared in the same way against the simulated data for ^18^FDG and Na^131^I, respectively.

### The influence of patient model morphology

In Table 4, the Ḣ^*^(10) per unit activity is given for each source organ, separately for a ^99m^Tc-HDP/MDP procedure for the different computational models described in Table 3 as well as for the ICRP female voxel phantom. The variation in ambient dose rate equivalent at 1 m from the different computational models is the highest (relative standard deviation of 26–30%) for the source regions ‘Urinary bladder content’ and ‘Kidneys’. For source region ‘Bone surface’ the relative standard deviation is 11%, while for the source region ‘Remainder’ almost no difference (~ 4%) was observed between the different computational models.

**Table 4:** Ḣ^*^(10) for each source region at 1 m, obtained by different computational models for a ^99m^Tc-MDP/HDP patient

	Ḣ^*^(10)_source organ_ [µSv/h]/MBq					
Source region	M0A^*^	M0B^*^	M0C^*^	M0D^*^	ICRP female phantom	Max / Min
Urinary bladder content	1.82E-02	1.27E-02	1.34E-02	1.15E-02	2.06E-02	1.8
Kidneys	1.29E-02	8.28E-02	1.12E-02	8.13E-03	1.61E-02	2.0
Remainder	1.41E-02	1.48E-02	1.42E-02	1.37E-02	1.50E-02	1.1
Bone surface	1.31E-02	1.11E-02	1.16E-02	9.63E-03	1.09E-02	1.4

^*^ Phantom code from [3]

**Fig. 8:**
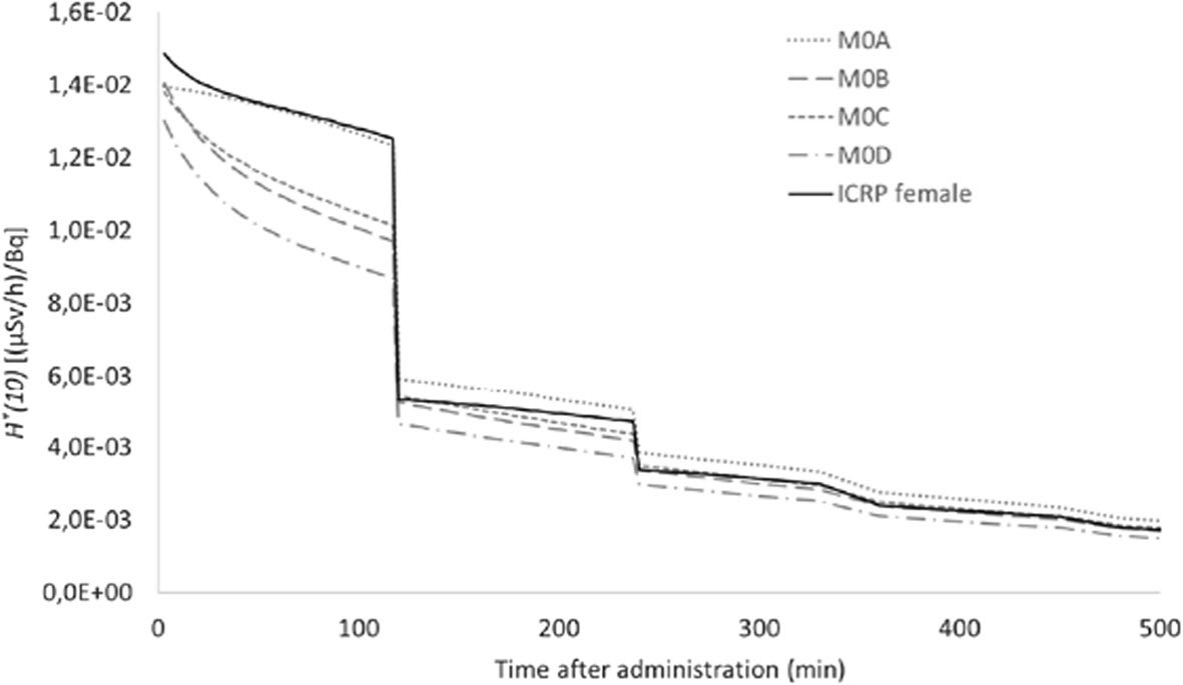
Comparison of computed Ḣ^*^(10)(t) post-injection at 1 m from different patient models for ^99m^Tc-HDP/MDP. Bladder voiding with 120 min interval p.a. has been considered

In Fig. 8, the Ḣ^*^(10)(t) is shown for all computational models with a relative standard deviation ranging from 4% for the earlier time points up to 16% for intermediate time points (i.e. 50–120 min p.a.) and around 10% for time points > 120 min p.a.

## Discussion

The validation of the computational framework has been performed successfully for a range of well-established MC codes. It was possible to implement two voxel computational models in several MC codes and the obtained ambient dose rate data per unit activity agreed well between the different codes (< 10% at 1 m). An extensive comparison between the different simulation parameters and input data from the range of MC codes used, demonstrated that the main contributors to the observed differences are variations in the way that the code users defined the distance of 1 m between the skin surface of both phantoms. We observed up to 10 cm variations between the different code users. A sensitivity study (not described here) with one specific code demonstrated that this variation of 10% resulted in differences in dose rate calculation less than 15% for ^99m^Tc and less than 10% for ^18^F and ^131^I. As a consequence, the relative difference between simulated data becomes larger if the distance between the phantoms becomes smaller, as the geometric variations will have a bigger impact (up to 16% for distances up to 30 cm).

Another sensitivity study (not described here) investigated the effect of performing the simulations with a simplified source spectrum (i.e. only including photons contributing more than 1% to the total number of disintegrations) against the complete energy spectrum of the isotope of interest. The differences were never higher than 6%.

There is also the uncertainty related to the choice of anthropomorphic models to represent both the NM patient and the exposed individual. Real NM patients and exposed individuals will never be an exact copy of these models. Although limited, the partial sensitivity study performed with different anthropomorphic computational models of similar height but varying weight showed that differences around 20% can be expected in dose rate values at 1 m from the patient for ^99m^Tc-HDP/MDP. Smaller differences are expected for ^18^F and ^131^I, as the energy of the emitted gammas is higher. The sensitivity study was performed with different computational male models which were already available to the investigators. These models have similar height as the ICRP female computational model, which was used in this study to represent the NM patient model. The aim of the sensitivity study was to investigate how much patient morphology affects the external dose rates, and to assess to what extent this could explain the differences observed between measured and simulated values of H^*^(10), and its outcome is independent on whether male or female models are used. A more extensive study on the influence of body morphology could be performed, including more phantom morphologies and postures, radiopharmaceuticals and distances. Working with flexible computational models as those developed by Lombardo et al*.* [17], which are more easily changed in size and posture, would facilitate such study.

The largest uncertainty in modelling the external dose rates from NM patients comes from the choice or availability of biokinetic models for the isotope under study. The available ICRP models are intended for reference individuals and not for describing patient-specific biokinetics, while the biokinetics from patients with a specific disease can vary significantly. Recently, efforts were done by Taprogge et al*.* [25] or are ongoing to update these ICRP models, with available patient data to obtain biokinetic information which is more representative for the average NM patient. The computational framework is built in such a way that the effect of new biokinetic information can easily be implemented and investigated without the need to repeat the Monte Carlo simulations for a specific patient-exposed individual configuration and radiopharmaceutical, as long as there are no new source regions in the new biokinetic models.

**Fig. 9:**
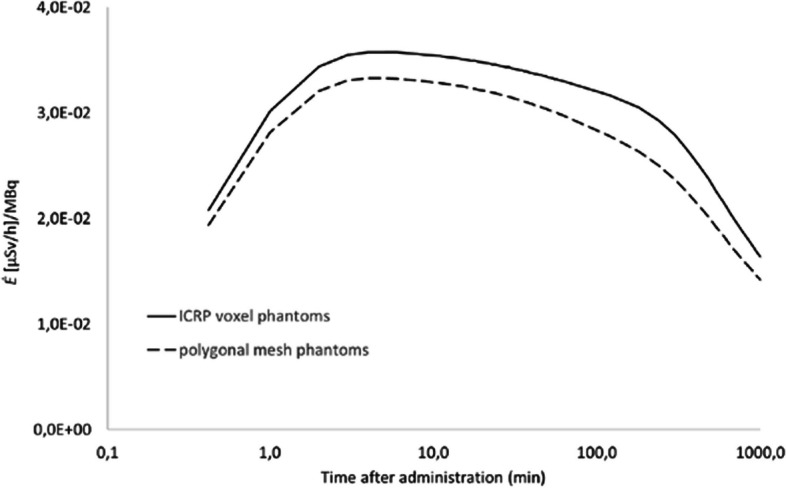
*Ė(t)* for Na^131^I comparing two types of computational models at 1 m distance: the ICRP voxel model [9]  and a polygonal mesh model from [17]

The computational framework in this study has been developed and tested for two diagnostic and one therapeutic radiopharmaceutical in simple configurations. This was done to benchmark the computational framework against experimental data which is mostly available for the above-mentioned radiopharmaceuticals and simplified configurations, i.e., at 1 m. As part of refining risk assessment studies, it will be much more interesting to investigate the external dose rates from NM patients in specific close-contact configurations e.g., breastfeeding patients and care activities of helpless patients, both for therapeutic and diagnostic procedures. This will be the next step in the study and for this purpose there will be the need to work with flexible computational models, like the tetrahedral mesh phantoms, such as the reference ICRP mesh phantoms [11] and the polygonal mesh phantoms, such as those developed by Lombardo et al. [17]. In Fig. 9, Monte Carlo simulations for Na^131^I with PHITS using the polygonal mesh phantoms from Lombardo et al*.* have been compared against those with the ICRP reference voxel phantoms. The effective dose rate per unit administered activity is calculated using the polygonal mesh phantoms for patient and exposed individual models facing each other at 1 m, showing on average results 14% lower (range: 6%—43% over the different source regions) for the polygonal mesh phantoms, compared against the ICRP reference voxel models. The observed differences, though are acceptable for the purpose of the study, considering uncertainties such as the choice of biokinetic model and the variety in sizes and morphology within a patient population.

## Conclusion

This study validated the computational framework that has been developed to assess the external dose rates from NM patients and the resulting exposure to an individual of a critical group. The validation has been performed successfully against experimental data of Ḣ^*^(10)(t) at 1 m from a NM patient. Next, several Monte Carlo codes agreed well in a more realistic scenario where both the NM patient and the exposed individual are represented by anthropomorphic computational models. The expected longer-term outcome from a more advanced computational framework, using flexible computational models and biokinetic data, is an online tool of effective dose rates per injected activity for a large variety of close-contact configurations, for a range of radiopharmaceuticals. Patient-specific information, such as whole-body retention data, could be included in such a tool to account for individualized features. Hospitals or regulatory bodies could use such a database/tool to evaluate the expected exposure to caregivers or the general public for specific scenarios as part of risk assessment studies. Together with the choice of appropriate dose constraints this would facilitate the setting of release criteria and patient restrictions. Moreover, besides determining the effective dose rates, also organ-specific dose rates per injected activity or fetus dose rates could be calculated separately.

## Data Availability

The datasets generated and/or analysed during the current study are uploaded (currently still in draft) in the ZENODO repository: [10.5281/zenodo.8414339].

## Abbreviations

^18^FDG: 2-Deoxy-2-[18F]fluoroglucose
GUM: Guide for the expression of uncertainty in measurements
ICRP: International commission on radiological protection
ICRU: International commission on radiation units and measurements
MC: Monte Carlo
Na^131^I: Sodium iodide
NM: Nuclear medicine
^99m^Tc-HDP/MDP: Hydroxymethylene diphosphonate/Methylene diphosphonate

## Declarations

### Liability statement

This paper summarizes the views of the EURADOS and of the EANM Radiation Protection Committee. It reflects recommendations for which the EANM cannot be held responsible. The recommendations should be taken into context of good practice of NM and do not substitute for national and international legal or regulatory provisions.
